# Spinal neuromodulation mitigates myocardial ischemia-induced sympathoexcitation by suppressing the intermediolateral nucleus hyperactivity and spinal neural synchrony

**DOI:** 10.3389/fnins.2023.1180294

**Published:** 2023-06-02

**Authors:** Siamak Salavatian, Yuki Kuwabara, Benjamin Wong, Jonathan R. Fritz, Kimberly Howard-Quijano, Robert D. Foreman, J. Andrew Armour, Jeffrey L. Ardell, Aman Mahajan

**Affiliations:** ^1^Department of Anesthesiology and Perioperative Medicine, University of Pittsburgh, Pittsburgh, PA, United States; ^2^Division of Cardiology, Department of Medicine, University of Pittsburgh Medical Center, Pittsburgh, PA, United States; ^3^Department of Anesthesiology and Perioperative Medicine, University of Pittsburgh Medical Center, Pittsburgh, PA, United States; ^4^Department of Physiology, University of Oklahoma Health Sciences Center, Oklahoma City, OK, United States; ^5^Cardiac Arrhythmia Center and Neurocardiology Research Program of Excellence, David Geffen School of Medicine at UCLA, Los Angeles, CA, United States

**Keywords:** spinal cord stimulation, neuromodulation, myocardial ischemia, autonomic nervous system, arrhythmias, spinal neural network

## Abstract

**Introduction:**

Myocardial ischemia disrupts the cardio-spinal neural network that controls the cardiac sympathetic preganglionic neurons, leading to sympathoexcitation and ventricular tachyarrhythmias (VTs). Spinal cord stimulation (SCS) is capable of suppressing the sympathoexcitation caused by myocardial ischemia. However, how SCS modulates the spinal neural network is not fully known.

**Methods:**

In this pre-clinical study, we investigated the impact of SCS on the spinal neural network in mitigating myocardial ischemia-induced sympathoexcitation and arrhythmogenicity. Ten Yorkshire pigs with left circumflex coronary artery (LCX) occlusion-induced chronic myocardial infarction (MI) were anesthetized and underwent laminectomy and a sternotomy at 4−5 weeks post-MI. The activation recovery interval (ARI) and dispersion of repolarization (DOR) were analyzed to evaluate the extent of sympathoexcitation and arrhythmogenicity during the left anterior descending coronary artery (LAD) ischemia. Extracellular *in vivo* and *in situ* spinal dorsal horn (DH) and intermediolateral column (IML) neural recordings were performed using a multichannel microelectrode array inserted at the T2-T3 segment of the spinal cord. SCS was performed for 30 min at 1 kHz, 0.03 ms, 90% motor threshold. LAD ischemia was induced pre- and 1 min post-SCS to investigate how SCS modulates spinal neural network processing of myocardial ischemia. DH and IML neural interactions, including neuronal synchrony as well as cardiac sympathoexcitation and arrhythmogenicity markers were evaluated during myocardial ischemia pre- vs. post-SCS.

**Results:**

ARI shortening in the ischemic region and global DOR augmentation due to LAD ischemia was mitigated by SCS. Neural firing response of ischemia-sensitive neurons during LAD ischemia and reperfusion was blunted by SCS. Further, SCS showed a similar effect in suppressing the firing response of IML and DH neurons during LAD ischemia. SCS exhibited a similar suppressive impact on the mechanical, nociceptive and multimodal ischemia sensitive neurons. The LAD ischemia and reperfusion-induced augmentation in neuronal synchrony between DH-DH and DH-IML pairs of neurons were mitigated by the SCS.

**Discussion:**

These results suggest that SCS is decreasing the sympathoexcitation and arrhythmogenicity by suppressing the interactions between the spinal DH and IML neurons and activity of IML preganglionic sympathetic neurons.

## 1. Introduction

The autonomic nervous system plays a critical role in regulating cardiac function (Armour, 2008). Myocardial ischemia by triggering the cardiac sensory neurons initiates the disruption in the cardiac autonomic nervous system (Zipes, 1990; Salavatian et al., 2019). This persistent afferent signaling by cardiac nociceptive afferents during myocardial ischemia causes an autonomic imbalance, manifested by sympathoexcitation, which leads to cardiac dysfunction and ventricular arrhythmias associated with sudden cardiac death (Zipes, 1990; Rubart and Zipes, 2005; Luqman et al., 2007; Fukuda et al., 2015; Rajendran et al., 2016).

During myocardial ischemic injury, triggered ischemia-sensitive cardiac afferent neurons transmit excitatory information to the dorsal horn of the spinal cord activating the spinal neural network (Dale et al., 2020; Omura et al., 2021). Activation of the ischemia-sensitive dorsal horn neurons leads to increased firing of the connected IML preganglionic sympathetic neuronal population. The excitatory signals from IML neurons to post-ganglionic neurons in the stellate and middle cervical ganglia results in increased sympathetic activity in the heart. This efferent sympathetic output to the heart can be exaggerated with myocardial injury, resulting in malignant ventricular tachyarrhythmias and potential for sudden cardiac death (Bourke et al., 2010; Fukuda et al., 2015). In chronic myocardial infarction (MI), significant neural remodeling at different levels of the cardiac neuraxis alters the global pattern of functional sympathetic innervation and increases cardiac arrhythmogenicity (Armour, 2004; Shen and Zipes, 2014; Vaseghi et al., 2014; Rajendran et al., 2016; Salavatian et al., 2022).

Cardiac neuromodulation therapies engage the underlying afferent and efferent neuronal projections for integrated reflex control of the heart. Neuromodulation via thoracic spinal cord stimulation (SCS) mitigates intrathoracic (intrinsic cardiac and intrathoracic extracardiac) reflexes while helping to preserve cardiac function (Odenstedt et al., 2011; Ardell, 2016; Howard-Quijano et al., 2017). SCS is also known to modify the capacity of cardiac sensory neurons to transduce the ischemic myocardium to the spinal cord dorsal horn neurons, thereby modulating spinal sympathetic efferent neuronal inputs to the heart (Ardell et al., 2019; Salavatian et al., 2019).

We have previously shown that SCS decreases myocardial ischemia-induced cardiac sympathoexcitation and ventricular arrhythmias (Howard-Quijano et al., 2017). However, it is not fully known how the SCS provides a therapeutic effect in the setting of myocardial infarction and myocardial ischemia to reduce cardiac sympathoexcitation and ventricular arrhythmias.

The primary purpose of this study was to determine how SCS modulates processing in the DH and IML spinal cord neuronal network during myocardial ischemia to mitigate cardiac arrhythmogenicity. We used a clinically relevant porcine model to mimic patients with prior MI who experience episodes of ventricular arrhythmias during the new onset of coronary artery ischemia. We hypothesized that SCS decreases myocardial ischemia-induced sympathoexcitation by suppressing intermediolateral nucleus hyperactivity and spinal neural synchrony.

## 2. Materials and methods

The Institutional Animal Care and Use Committee (IACUC) at the University of Pittsburgh approved the study protocol. All experiments were performed in compliance with the National Institution of Health Guide for the Care and Use of Laboratory Animals.

### 2.1. Left circumflex coronary artery MI creation

**FIGURE 1:**
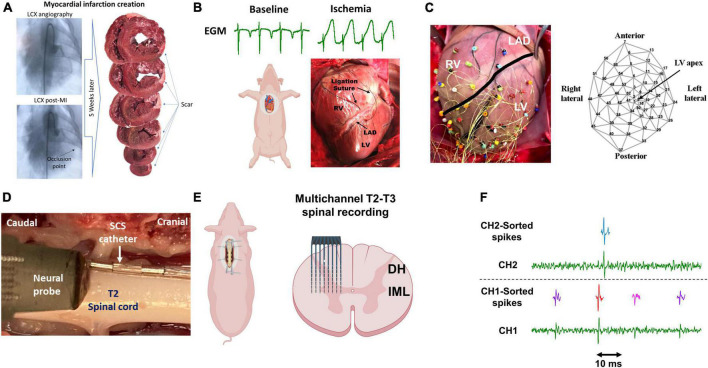
Experimental protocol and methodology. **(A)** Chronic myocardial infarction was induced by injection of the microsphere in the left circumflex coronary artery (LCX). The scar zone was measured at the terminal experiments 4–5 weeks later. **(B)** Following sternotomy, acute myocardial ischemia was performed by ligating the left descending anterior coronary artery (LAD) and was confirmed by the ST-elevation on the epicardial electrograms (EGM). RV/LV: right/left ventricle. **(C)** 56-electrodes epicardial sock was used to record the electrogram and measure the activation recovery interval and dispersion of the repolarization. **(D)** A representative image of the location of the neural probe and the spinal cord stimulation (SCS). **(E)** A Multichannel multi-shank microelectrode array was used to record the activity of spinal cord neurons in the dorsal horn (DH) and intermediolateral column (IML) regions. **(F)** Spike sorting was performed to identify and classify the spiking activity of each neuron. Activities from 3 neurons (purple, red, pink) and one neuron (green) were identified based on their action potential shapes on channel 1 (CH1) and channel 2 (CH2), respectively. Parts of this figure were created with BioRender.com.

Ten Yorkshire pigs (four males and six females, mean ± SD, 31.2 ± 2.4 kg) were used. All animals were allowed to get acclimated for at least 5 days before survival surgery in the animal facility. Animals were sedated (tiletamine-zolazepam, 4 mg/kg IM + Xylazine, 2 mg/kg IM), intubated, and ventilated under general anesthesia using 1−3% of isoflurane with hemodynamic monitoring. Left circumflex coronary artery (LCX) MI was created as previously described (Salavatian et al., 2022). Under fluoroscopic guidance, an 8F AL2 guide catheter (Boston Scientific, Marlborough, MA, USA) was advanced from the femoral artery to the ostium of the left main coronary artery. A 3 mm percutaneous transluminal angioplasty balloon catheter (Abbott, Chicago, IL, USA) was advanced over a balance middleweight universal guidewire (Abbott, Chicago, IL, USA) into the LCX and positioned at the distal LCX. The balloon was inflated, and 3 mL of polystyrene microspheres (Polybead 90.0 μm, Polysciences, Warrington, PA, USA) followed by 1−2 mL of normal saline were injected through the lumen of the catheter. The balloon was then deflated and removed. MI was confirmed by the presence of ST-elevation or T-wave inversion on electrocardiogram (ECG) and coronary angiography showing a lack of flow in the artery (Figure 1). After the procedure, ECG and blood pressure were monitored until the pig remained hemodynamically stable for at least 20 min before extubation. Immediate external defibrillation was performed if the animal developed sustained ventricular tachycardia or fibrillation. After extubating, all animals were further monitored until they could ambulate.

### 2.2. Terminal experiment – animal preparation

After 4−5 weeks of MI, the terminal experiment was performed and animal preparations were conducted as previously reported (Salavatian et al., 2019; Omura et al., 2021). Animals (*n* = 10, 50.7 ± 6.5 kg) were sedated with Telazol (4 mg/kg, intramuscular) and Xylazine (2 mg/kg, intramuscular), and mechanically ventilated by tracheal intubation. General anesthesia was induced and maintained with inhaled 2−5% of isoflurane during surgical preparation. Heart rate and ECG were monitored throughout the experiment using a Prucka CardioLab system (GE Healthcare, Fairfield, CT, USA). The carotid artery was catheterized for blood pressure monitoring. In addition, jugular veins were cannulated for intravenous saline infusion (10 ml/kg) and drug administration. Arterial blood gas was checked every hour with adjustment of ventilation if needed to maintain acid-base equilibrium. Body temperature was maintained by external warmers. Dorsal spinal laminectomy was done in a prone position to expose the spinal cord, then median sternotomy was then performed supine to expose the heart. General anesthesia was transitioned to intravenous alpha-chloralose (50 mg/kg initial bolus followed by a 20 mg/kg/h continuous infusion), which limits the impact on the autonomic nervous system and cardiac electrophysiology (Luckl et al., 2008; Omura et al., 2021). The experimental protocol was conducted in the left lateral position to record spinal cord neural activity and cardiac electrophysiology with acute myocardial ischemia. The depth of anesthesia was assessed throughout the experiments by monitoring corneal reflexes, jaw tone, and hemodynamic indices. All animals were euthanized by inducing ventricular fibrillation via potassium chloride administration under deep anesthesia. The heart was excised and chronic infarct size was measured with triphenyl tetrazolium chloride staining as previously reported (Takagawa et al., 2007).

### 2.3. Recording of spinal cord neuronal activity

Laminectomy was performed at T1-T4 levels of the spinal cord to expose the T2-T3 level and provide enough space for the insertion of the neural probe. The animal was placed in the right lateral position and following a small opening in the dura, a high-density two-dimensional penetrating microarray (64 electrode recording sites; NeuroNexus, Ann Arbor, MI, USA) probe, mounted on a micromanipulator, was inserted in the spinal cord at the T2-T3 level (Figure 1). The neural probe was inserted into the spinal cord to record the neuronal activity of the DH and IML neurons (and not the axons due to the high impedance of the headstage and filtering) *in vivo* and *in situ*. Recorded signals were amplified, digitized, and filtered (300 Hz – 3 kHz) using the SmartBox acquisition system (NeuroNexus, Ann Arbor, MI, USA).

### 2.4. Acute myocardial ischemia

We created acute myocardial ischemia as previously described (Howard-Quijano et al., 2021). Briefly, a Prolene suture was placed around the left anterior descending coronary artery (LAD) below the second diagonal branch of the LAD. The suture was led through a short polyethylene tubing segment, which was then used to ligate the coronary artery to induce cardiac ischemia. LAD ischemia was confirmed with the existence of ST elevations (Figure 1). Short LAD ischemia (3 min) was induced before and 1 min after SCS.

### 2.5. Cardiac mechanical and nociceptive stimuli

To identify the mechanosensitive and nociceptive neurons, a set of mechanical and nociceptive stimuli were performed. For the mechanical stimuli, the right and left anterior ventricular epicardium were touched for 15 s. To identify nociceptive neurons, ventricular (anterior) epicardial nociceptive stimuli were applied for 1 min using a gauze soaked in bradykinin (10 μM) or capsaicin (1 μM). The neurons that responded to both mechanical and nociceptive stimuli, were identified as multimodal neurons. In this study, we only analyzed the mechanical, nociceptive, and multimodal neurons that also responded to the myocardial ischemia (i.e., ischemia-sensitive mechanical, nociceptive, or multimodal neurons).

### 2.6. Hemodynamic measurements

Electrocardiogram was continuously recorded on the Prucka CardioLab system (GE Healthcare, Fairfield, CT, USA). A 5 French Millar Mikro-Tip pressure transducer catheter (SPR-350) was inserted into the left ventricle via the left carotid artery and the continuous data was recorded in an MPVS Ultra Pressure-Volume Loop System (Millar Instruments, Houston, TX, USA) throughout the experiment. Left ventricular systolic function was evaluated by the maximum rate of pressure change (dp/dt max).

### 2.7. Cardiac electrophysiological measurements

Epicardial 56-electrode nylon mesh sock electrodes were placed around the heart and unipolar electrograms (0.05−500 Hz) were measured using a Prucka CardioLab electrophysiology mapping system (GE Healthcare, Fairfield, CT, USA) (Figure 1). We assessed the activation recovery interval (ARI), which has been shown as a surrogate of local action potential duration (Millar et al., 1985). ARIs were calculated with customized software (iScalDyn, University of Utah, Salt Lake City, UT, USA), as previously described (Howard-Quijano et al., 2021). Ischemic regions were defined based on the electrodes with ST elevations and each electrogram with ST-segment changes was analyzed by semiautomated accepted software as well as manually following the guidelines described by Haws and Lux (1990). Global epicardial dispersion of repolarization (DOR) which is associated with a heterogeneity of repolarization time and an increased risk for ventricular arrhythmias, was also analyzed (Vaseghi et al., 2012).

### 2.8. Spinal cord stimulation

A spinal cord stimulating lead with 8 electrodes was inserted in the epidural space with the leads located at the left T1-T4 spinal cord level and the most cranial pole of the lead at T1 (Figure 1). Current controlled stimulation (model S88 stimulator; Grass Instruments, Quincy, MA, USA) was delivered at 1 kHz, 0.03 ms pulse width for 30 min. Stimulation currents were set at 90% of a motor threshold, which was determined by increasing stimulus intensity at 2 Hz and 0.4 ms pulse width until muscle contractions were observed in the shoulder.

### 2.9. Neuronal activity analysis

#### 2.9.1. Spike sorting

Extracellular action potentials were recorded using a multichannel microelectrode array. Each electrode is capable of recording the activity of multiple neurons simultaneously. To be able to follow the activity of each neuron and its response to various stimuli and interventions, spike sorting was performed to identify and classify the neurons (Figure 1). Action potentials with a signal-to-noise ratio of greater than 2:1 were detected and were classified using the principal component analysis and cluster on measurements techniques in the Spike2 software program (Cambridge Electronics Design, United Kingdom). Artifacts were identified as simultaneously occurring waveforms on more than 2 adjacent electrodes as based on the specification of the multichannel electrode, it is not possible to record the neural activity from a single neuron on multiple electrodes. The artifacts were removed before the waveform classification.

#### 2.9.2. Identification of intermediolateral neurons

Sympathetic control of the heart is initiated by the preganglionic sympathetic neurons in the IML region of the spinal cord. These preganglionic neurons project their axons into the paravertebral chain to post-ganglionic neurons contained with Stellate and Middle Cervical Ganglia. Stimulation of the paravertebral chain activates the IML neurons antidromically. To identify the IML neurons at the T2 level of the spinal cord, the T2 paravertebral chain was stimulated using a Grass S88 Stimulator (Grass Instruments, Quincy, MA, USA). To set the stimulation current intensity, first, a current threshold to induce a 10% increase in the blood pressure by stimulation at 4 Hz and 4 ms was performed. The actual stimulation to identify the IML neurons was delivered for 1 min at 1 Hz, 1 ms, and at the current threshold defined above. The neurons that had more than 60% one-to-one firing response within the 50 ms of the T2 stimulation pulse and with consistent delay were classified as the IML neurons (Dale et al., 2020; Omura et al., 2021). These neurons were recorded from an electrode zone that was in the IML region. All other recorded neurons were labeled as DH neurons.

#### 2.9.3. Assessment of spinal neural response to myocardial ischemia

To assess the response of spinal neurons to the myocardial ischemia prior to and after SCS, the firing rate of each neuron was compared between the 1 min pre-LAD vs. during the 3 min LAD. Neurons that had a significant increase in the firing rate during the LAD ischemia prior to SCS, were identified as ischemic-sensitive neurons. To evaluate the efficacy of SCS in reducing the firing response of these neurons to the LAD ischemia, the activity of these neurons was followed during the LAD post-SCS to investigate their response change to LAD ischemia pre- vs. post-SCS.

#### 2.9.4. Neural network interactions

In this study, we have used the measure of synchrony method that was previously developed to analyze the neural network interactions in the intrinsic cardiac nervous system (Longpre et al., 2014). In this method, we have calculated the normalized jitter-based synchrony index (SI), (Agmon, 2012) among the pair of neurons to assess the level and statistical significance of synchrony between spinal neurons. Neuron synchronization was analyzed by pair of neurons (one as a reference and one as a target). Coincidence firing between a reference and a target neuron occurred when both neurons fire within a time window of 40 ms. Using the above coincidence firing definition, some neurons with a high firing rate would cause false positive coincidence. These random coincidences are identified and the final SI is normalized based on these random coincidences that might happen by chance.

### 2.10. Statistical analysis

The sample size of the study was selected based on our previous published work with a similar neural recording protocol. To identify a statistically significant change in the firing rate of each neuron at different time intervals, a statistical test derived from the Skellam distribution was used (Shin et al., 2010) which was previously used for spinal and peripheral neural firing analysis (Beaumont et al., 2013; Salavatian et al., 2019; Omura et al., 2021). A statistically significant change in the activation recovery interval and dispersion of repolarization between baseline and during LAD was assessed by the paired *t*-test. For the statistical analysis of the neural firing data, since the data were not normally distributed, the Friedman test with Dunn’s multiple comparisons test was performed to investigate the change in firing rate during LAD and the reperfusion. The Kruskal–Wallis test was used to compare the firing rate of mechanosensitive, nociceptive, and multimodal neurons. Wilcoxon test was used to compare the non-paramteric paired synchrony changes. Statistical analyses were performed using Prism (version 8, GraphPad Software Inc., San Diego, CA, USA) and a *P*-value of 0.05 was considered statistically significant. Data are reported as mean ± SE.

## 3. Results

We have induced cardiac insult in the different regions of the heart, where chronic MI was percutaneously induced in the left lateral region by injecting microspheres into the LCX coronary artery to create a patchy infarct, and acute myocardial ischemia was induced by ligating the LAD coronary artery. All ten pigs with the MI survived for 4−5 weeks until the terminal experiment and the MI size at the terminal experiment was 10.8 ± 5.5% within the left ventricle. Acute ischemic insult measured by the number of ST elevation leads was comparable between ischemia at pre- and post-SCS. The hemodynamic changes during SCS were not significant (Table 1). No difference was observed between the hemodynamic response of ischemia and reperfusion pre-SCS vs. post-SCS (Table 2).

**TABLE 1:** Hemodynamic response to spinal cord stimulation (SCS).

SCS	Baseline	During SCS	Post-SCS
HR (beat/min)	106.5 ± 7.1	107.2 ± 6.5	104.5 ± 7.7
LVSP (mmHg)	108.4 ± 8.2	111.1 ± 7.8	106.5 ± 7.5
dp/dt max (mmHg/s)	1256.0 ± 100.1	1241.0 ± 92.34	1243.0 ± 99.33
dp/dt min (mmHg/s)	−1350.0 ± 106.0	−1368.0 ± 101.1	−1332.0 ± 103.2

**TABLE 2:** Delta change in hemodynamic parameter in response to ischemia and reperfusion.

	LAD pre-SCS	LAD post-SCS	Rep. pre-SCS	Rep. post-SCS
HR (beat/min)	2.42 ± 0.8	0.88 ± 0.83	−2.4 ± 2.0	−1.2 ± 0.5
LVSP (mmHg)	−8.0 ± 2.4	−5.1 ± 1.8	1.4 ± 1.9	4.2 ± 2.0
dp/dt max (mmHg/s)	−111.8 ± 22.1	−74.74 ± 19.6	134.5 ± 31.7	109.7 ± 27.04
dp/dt min (mmHg/s)	178.3 ± 17.73	120.0 ± 19.8	−53.25 ± 34.31	−86.71 ± 29.32

### 3.1. Effect of myocardial ischemia on activation recovery interval and dispersion of repolarization

**FIGURE 2:**
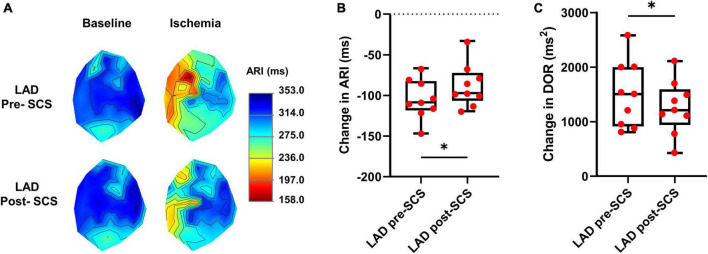
Effect of myocardial ischemia on activation recovery interval and dispersion of repolarization. **(A)** Representative activation recovery interval (ARI) map during the left anterior descending coronary artery (LAD) ischemia pre- and post-spinal cord stimulation (SCS). **(B)** ARI shortening during LAD ischemia was reduced after SCS, indicating less sympathoexcitation during myocardial ischemia post-SCS (*N* = 9 pigs). **(C)** SCS is decreasing the arrhythmogenicity by reducing the augmentation of dispersion of repolarization (DOR) during myocardial ischemia (*N* = 9 pigs). *Denotes *P* < 0.05.

The cardiac electrophysiological data of nine pigs were analyzed and the data from one pig was removed due to the poor quality of the cardiac electrophysiological signal. The ARI is a surrogate for the action potential duration and in this study, we have measured the ARI at baseline and during the LAD to evaluate the magnitude of sympathoexcitation caused by the myocardial ischemia. Sympathetic activation is associated with shortened activation recovery interval duration and increased dispersion of repolarization (Yamakawa et al., 2016). ARI in the ischemic zone was shortened during the myocardial ischemia (baseline:314.3 ± 13.6 ms, LAD: 210.1 ± 7.2 ms, *P* < 0.0001) indicating the sympathoexcitation (Figure 2). The level of arrhythmogenicity was assessed by measuring the DOR at baseline and during myocardial ischemia. The DOR was increased during myocardial ischemia (baseline: 443.9 ± 93.05 ms^2^ to LAD: 1941 ± 213.8 ms^2^, *P* < 0.0001) showing increased arrhythmogenicity during the LAD ischemia.

### 3.2. Spinal cord stimulation effect on sympathoexcitation and arrhythmogenicity

To evaluate the effect of SCS on sympathoexcitation and arrhythmogenicity, the extent of ARI shortening and DOR augmentation were compared during the LAD ischemia before and after 30 min of SCS. SCS suppressed the sympathoexcitation during LAD post-SCS (ARI shortening: pre-SCS: −104.10 ± 8.04 ms; post-SCS: −87.80 ± 8.75 ms, *P* = 0.028) (Figure 2). The arrhythmogenicity was also reduced by SCS, manifested by less augmentation in the DOR during LAD post-SCS (DOR change: pre-SCS: 1497 ± 201.9 ms^2^; post-SCS: 1260 ± 164.7 ms^2^, *P* = 0.024 vs. pre-SCS) (Figure 2).

### 3.3. Effect of spinal cord stimulation on spinal neural response to myocardial ischemia

**FIGURE 3:**
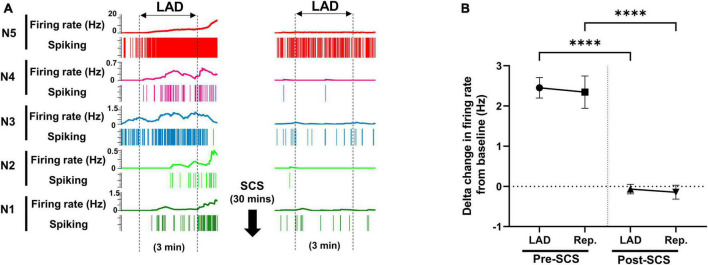
Effect of spinal cord stimulation on firing response of spinal neurons to myocardial ischemia. **(A)** Representative neural activity of five spinal neurons during the left anterior descending coronary artery (LAD) ischemia pre- and post-spinal cord stimulation (SCS). The activity of these five neurons increases during the ischemia, however, they have a different temporal response to the myocardial ischemia and reperfusion. A 30-s averaging window was used for the firing rate data in this panel. **(B)** The firing rate change (firing rate during LAD or Rep. - firing rate at baseline) of spinal neurons (*n* = 257) to LAD and reperfusion (Rep.) is shown pre and post-SCS. Negative values, indicate the decreasing firing rate. Data is presented as mean ± SE. ****Denotes adjusted *P*-value < 0.0001 after Dunn’s multiple comparisons tests.

Using the multichannel microelectrode array, 2,758 spinal neurons were recorded. Spinal neurons (*n* = 257) that were activated by myocardial ischemia were labeled as ischemia-sensitive neurons. IML neurons (*n* = 29) and DH neurons (*n* = 228) were identified, and their firing activities were followed during the whole protocol. The activity of ischemia-sensitive spinal neurons (*n* = 257) was evaluated during the myocardial ischemia post-SCS (Figure 3). The ischemia-sensitive neurons’ response to the LAD ischemia and reperfusion was reduced post-SCS (pre-SCS: LAD: 2.45 ± 0.25 Hz, reperfusion: 2.34 ± 0.402 Hz; post-SCS: LAD: −0.07 ± 0.11 Hz, *P* < 0.0001 vs. LAD pre-SCS, reperfusion: −0.14 ± 0.17 Hz, *P* < 0.0001 vs. reperfusion pre-SCS). The response of IML neurons (Pre-SCS: LAD: 3.50 ± 0.53 Hz, reperfusion: 4.19 ± 1.24 Hz; Post-SCS: LAD: 0.705 ± 0.67 Hz, *P* = 0.0027 vs. LAD pre-SCS, reperfusion: 0.29 ± 0.96 Hz, *P* = 0.0027 vs. reperfusion pre-SCS) and DH neurons (Pre-SCS: LAD: 2.27 ± 0.27 Hz, reperfusion: 2.29 ± 0.39 Hz; Post-SCS: LAD: −0.16 ± 0.104 Hz, *P* < 0.0001 vs. LAD pre-SCS, reperfusion: −0.20 ± 0.16 Hz, *P* < 0.0001 vs. reperfusion pre-SCS) to myocardial ischemia and reperfusion was also blunted after the SCS.

### 3.4. Response of effect of spinal cord stimulation on different types of DH neurons

**FIGURE 4:**
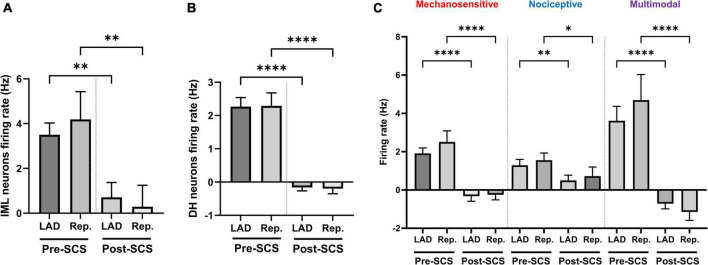
Effect of spinal cord stimulation on the intermediolateral and dorsal horn neuronal hyperactivity during myocardial ischemia. Spinal cord stimulation (SCS) suppressed the response of panel **(A)** intermediolateral column (IML) neurons (*n* = 28, the activity of 1 neuron was removed based on the outlier criteria by ROUT method and *Q* = 0.1%), **(B)** dorsal horn (DH) neurons (*n* = 228), and **(C)** ischemia-sensitive mechanosensitive (*n* = 49), nociceptive (*n* = 37) and multimodal neurons (*n* = 59) to the left anterior descending coronary artery (LAD) ischemia and reperfusion (Rep.). Data is presented as mean ± SE and *, **, ****denotes *P* < 0.05, *P* < 0.01, and *p* < 0.0001, respectively.

Mechanosensitive (*n* = 49), nociceptive (*n* = 37), and multimodal (*n* = 59) ischemia-sensitive DH neurons were characterized. To investigate if the response of mechanosensitive, nociceptive, and multimodal neurons to LAD ischemia is affected differently by the SCS, we have evaluated the effect of SCS on the firing response of each of these groups to the LAD ischemia and reperfusion (Figure 4). SCS mitigated the response of mechanosensitive (Pre-SCS: LAD: 1.91 ± 0.29 Hz, reperfusion: 2.51 ± 0.60 Hz; Post-SCS: LAD: −0.32 ± 0.26 Hz, *P* < 0.0001 vs. LAD pre-SCS, reperfusion: −0.25 ± 0.26 Hz, *P* < 0.0001 vs. reperfusion pre-SCS), nociceptive (Pre-SCS: LAD: 1.29 ± 0.30 Hz, reperfusion: 1.55 ± 0.38 Hz; Post-SCS: LAD: 0.50 ± 0.28 Hz, *P* = 0.009 vs. LAD pre-SCS, reperfusion: 0.72 ± 0.47 Hz, *P* = 0.02 vs. reperfusion pre-SCS) and multimodal (Pre-SCS: LAD: 3.62 ± 0.75 Hz, reperfusion: 4.70 ± 1.34 Hz; Post-SCS: LAD: −0.72 ± 0.27 Hz, *P* < 0.0001 vs. LAD pre-SCS, reperfusion: −1.15 ± 0.44 Hz, *P* < 0.0001 vs. reperfusion pre-SCS) neurons to the myocardial ischemia.

### 3.5. Neural synchrony response alterations among DH-DH and DH-IML neurons to SCS

**FIGURE 5:**
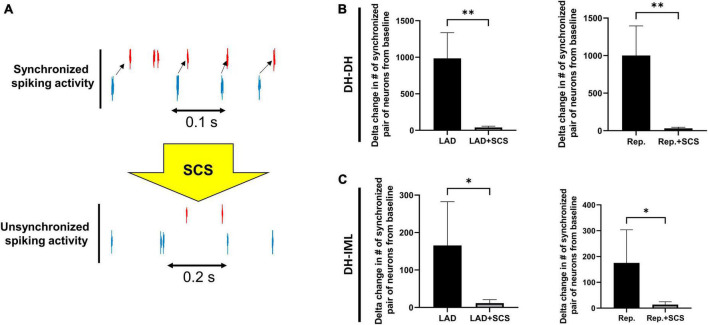
Effect of spinal cord stimulation on spinal neuronal synchrony during myocardial ischemia. **(A)** Representative activity of two neurons when they have synchronized and unsynchronized activity. **(B)** The augmentation in the neuronal synchrony among DH-DH neurons (*n* = 2668, *n* = 2668) during left anterior descending coronary artery (LAD) ischemia and reperfusion (Rep.) was suppressed by the spinal cord stimulation (SCS). **(C)** The SCS showed a similar effect on suppressing the synchrony among DH-IML pair of neurons (DH: *n* = 2668, IML: *n* = 90) synchrony during LAD, and reperfusion. *Denotes *P* < 0.05, **denotes *P* < 0.01.

The neural synchrony between the DH and IML neurons was evaluated during LAD ischemia, and reperfusion pre- and post-SCS (Figure 5). The LAD ischemia-induced augmentation in neuronal synchrony between DH-DH (pre-SCS:985.30 ± 351.39, vs. post-SCS: 39.60 ± 17.26, *p* = 0.004) and DH-IML (pre-SCS:165.60 ± 116.76, vs. post-SCS: 11.50 ± 9.45, *p* = 0.02) pairs of neurons were mitigated by the SCS. SCS also suppressed the reperfusion-induced augmentation in neuronal synchrony between DH-DH (pre-SCS: 1001.60 ± 393.85, vs. post-SCS: 31.30 ± 15.28, *p* = 0.008) and DH-IML (pre-SCS:175.20 ± 128.41, vs. post-SCS: 13.40 ± 11.49, *p* = 0.02).

## 4. Discussion

We have developed a pre-clinical large animal model that mimics a clinical situation in patients with prior myocardial infarction who experiences episodes of ventricular arrhythmias due to the new onset of acute coronary artery ischemia. This is the first study, to our knowledge, that shows the therapeutic effect of high-frequency SCS on cardiac sympathoexcitation and arrhythmogenicity during acute myocardial ischemia in the presence of chronic MI.

Our major findings are as follows: in a chronic MI heart (1) 3 min of acute myocardial ischemia shortened ARI, which was mitigated by preemptive SCS therapy, (2) acute myocardial ischemia increased DOR, which was mitigated by preemptive SCS therapy. In addition, (3) IML sympathetic preganglionic neural firing rate increased with acute myocardial ischemia and reperfusion injury, which was blunted with preemptive SCS (4) the firing response of the mechanosensitive, nociceptive, and multimodal neurons to myocardial ischemia and reperfusion were mitigated by preemptive SCS therapy, (5) myocardial ischemia and reperfusion increased the synchrony among the DH-DH and DH-IML neurons and this effect was blocked by SCS.

### 4.1. Effect of SCS on cardiac electrophysiology

We and others have previously shown that SCS can decrease cardiac sympathoexcitation and arrhythmogenicity induced by myocardial ischemia in a healthy heart (Lopshire et al., 2009; Odenstedt et al., 2011; Howard-Quijano et al., 2017, 2021). While the underlying mechanisms are not exactly elucidated yet, we have found that SCS increases an inhibitory neurotransmitter GABA within the thoracic spinal cord, which may decrease an efferent outflow to the heart, thus reducing cardiac sympathoexcitation and ventricular arrhythmias (Howard-Quijano et al., 2023). In this study, we have shown the effectiveness of the high-frequency SCS in reducing acute myocardial ischemia-induced sympathoexcitation and arrhythmogenicity in subjects with prior chronic myocardial infarction. Although we used the low-frequency (50 Hz) SCS in our previous studies and showed the efficacy of SCS to prevent arrhythmogenicity in pre-clinical models, a high-frequency of SCS such as 1 kHz may provide more superior effects on cardiac pathophysiology as a randomized controlled trial comparing the high-frequency and conventional stimulation demonstrated the superiority of the high-frequency stimulation for the treatment in the pain field (Kapural et al., 2015).

### 4.2. Impact of SCS on spinal neural firing rate during myocardial ischemia

Spinal cord stimulation as a bioelectric neuromodulation engages central neuronal reflexes (Ardell, 2016) to influence ischemia-sensitive spinal cord neurons with regard to both pain perception (Qin et al., 2008) and modulating central-mediated sympathoexcitation (Ding et al., 2008). SCS can mitigate myocardial ischemia-induced reflex activation of cardiac neural networks (Foreman et al., 2000; Ardell et al., 2009). These reflexes are known to exhibit memory, with neural network protective effects extending up to 1 h after SCS (Armour et al., 2002). We have previously shown that acute myocardial ischemia activated cardiac-related DRG neurons and SCS decreased basal activity of such responsiveness to transient myocardial ischemia in the thoracic DRG in a healthy heart (Salavatian et al., 2019).

In this study, we showed that ischemia-activated neurons in the dorsal horn and IML regions of the spinal cord were suppressed by high-frequency SCS, suggesting that SCS mitigated cardiac efferent output from the spinal cord to the heart, which would contribute to lower sympathetic excitation and reduce the risk of ventricular arrhythmias. Interestingly, this mitigation of neuronal firing was also seen during the reperfusion period, which causes tremendous pathophysiological damage to the heart in various ways (Soares et al., 2019), thus indicating that SCS has a memory effect to reduce cardiac sympathoexcitation even after cessation of SCS.

Moreover, we have shown the impact of SCS on blunting the LAD ischemia-induced neuronal hyperexcitation is not significantly different among the ischemia-sensitive mechanical, nociceptive, and multimodal neurons, suggesting that high-frequency SCS is not selective in suppressing just the neural activity of ischemia-sensitive neurons.

### 4.3. Impact of SCS on spinal neural synchrony during myocardial ischemia

We have previously shown that myocardial ischemia increases the synchrony among the dorsal horn and IML pair of neurons (Dale et al., 2020; Omura et al., 2021). We hypothesize that this increase in synchrony and augmented neuronal communication occurs because of the higher processing needs of the spinal cord during myocardial ischemia. In this study, we showed that this augmented synchrony during both myocardial ischemia and reperfusion is decreased by the SCS. It is important to mention that in this study, we have used a normalized synchrony method. This method normalizes the synchrony index in such a way that the random firing of two high-firing neurons does not necessarily result in a high synchrony index among these neurons.

Myocardial ischemia activates cardiac afferent inputs to the thoracic spinal cord through dorsal root ganglia (Malliani et al., 1981; Fukuda et al., 2015), and it associates with increased coordination within dorsal horn neurons and between dorsal horn to sympathetic preganglionic neurons in the thoracic spinal cord (Dale et al., 2020). In this study, we have also observed the augmentation in the synchronized pairs of neurons in the spinal cord afferent processing sites (between DH and DH neurons) and between the spinal afferent and efferent processing sites (between DH and IML neurons). This suggests that during the myocardial ischemia-reperfusion injury, there is a high level of spinal processing which is manifested by a high firing rate in the DH and high synchrony among the DH-DH neurons and high synchrony among the DH-IML neurons, which leads to the increased firing rate of IML neurons and sympathoexcitation.

### 4.4. Clinical implications

The clinical trials of the SCS for heart diseases have been equivocal. In the heart failure patients, while the DEFEAT-HF trial showed no improvement in left ventricular end-systolic volume index, peak oxygen uptake, N-terminal pro-B-type natriuretic peptide [NT-proBNP] endpoints (Zipes et al., 2016), the SCS HEART trial demonstrated improvement in New York Heart Association (NYHA) classification, quality of life, left ventricular ejection fraction, left ventricular volume, and peak oxygen consumption (Tse et al., 2015). This equivocal outcome could be a result of not selecting optimized stimulation parameters due to the lack of understanding of the mechanism of the SCS in the modulation of the spinal neural network. In this study, we have evaluated the efficacy of SCS in mitigating the myocardial ischemia-induced sympathoexcitation and arrhythmogenicity in the subjects with myocardial infarction to evaluate if the SCS therapy after a chronic myocardial infarction can provide cardioprotection against the new myocardial ischemic events. The candidates for this therapy would be the patients who have developed a chronic MI and are seeking to find a therapy to decrease the chance of reoccurrence of the fatal ischemic-induced ventricular arrhythmias.

### 4.5. Limitations

We induced chronic MI into the LCX coronary artery in order to allow for a second acute myocardial ischemia into the LAD coronary artery. It is possible that chronic myocardial infarction into the LAD region creates a different arrhythmogenicity and neuronal remodeling in the cardio-spinal neural pathway. General anesthesia can suppress neuronal activity but is necessary to conduct the animal experiment. Isoflurane was switched to alpha-chloralose to minimize the effects of isoflurane on spinal neural activity. Since this study was performed in an anesthetized animal at rest, in which the sympathetic tone is low, we could not evaluate the effect of SCS on the sympathetic function without the myocardial ischemia. Further studies in normal awake animals are needed to evaluate the impact of the SCS on normal sympathetic function. Another limitation of this study is that since the memory effect of 30 min- SCS was not known, sham ischemia was always performed before the ischemia + SCS.

## 5. Conclusion

Myocardial ischemia induces sympathoexcitation by increasing the IML activity which is caused by the activation of DH neurons and increased spinal neural synchrony between DH and IML neurons. SCS mitigates the adverse effect of myocardial ischemia by suppressing spinal neural synchrony and IML hyperactivity.

## Data availability statement

The raw data supporting the conclusions of this article will be made available by the authors, without undue reservation.

## Ethics statement

This animal study was reviewed and approved by the Institutional Animal Care and Use Committee (IACUC).

## Author contributions

SS, JAA, JLA, RF, and AM conceived and designed the research. SS, YK, BW, and JF conducted the experiments and analyzed the data. SS, YK, JAA, JLA, RF, KH-Q, and AM interpreted the results of the experiments. SS and YK drafted the manuscript and prepared the figures. All authors reviewed and approved the final version of the manuscript.
